# Bioinstructive Layer-by-Layer-Coated Customizable 3D Printed Perfusable Microchannels Embedded in Photocrosslinkable Hydrogels for Vascular Tissue Engineering

**DOI:** 10.3390/biom11060863

**Published:** 2021-06-10

**Authors:** Cristiana F. V. Sousa, Catarina A. Saraiva, Tiago R. Correia, Tamagno Pesqueira, Sónia G. Patrício, Maria Isabel Rial-Hermida, João Borges, João F. Mano

**Affiliations:** CICECO–Aveiro Institute of Materials, Department of Chemistry, University of Aveiro, Campus Universitário de Santiago, 3810-193 Aveiro, Portugal; cristiana.sousa@ua.pt (C.F.V.S.); catarina.a.m.saraiva@gmail.com (C.A.S.); trcorreia@ua.pt (T.R.C.); tamagno1992@gmail.com (T.P.); sgpatricio@ua.pt (S.G.P.); mariaisabel.rial@usc.es (M.I.R.-H.)

**Keywords:** biocompatible polymers, 3D printing, layer-by-layer assembly, perfusable multilayered microchannels, natural-origin hydrogels, endothelial cells, prevascularized networks, modular tissue engineering

## Abstract

The development of complex and large 3D vascularized tissue constructs remains the major goal of tissue engineering and regenerative medicine (TERM). To date, several strategies have been proposed to build functional and perfusable vascular networks in 3D tissue-engineered constructs to ensure the long-term cell survival and the functionality of the assembled tissues after implantation. However, none of them have been entirely successful in attaining a fully functional vascular network. Herein, we report an alternative approach to bioengineer 3D vascularized constructs by embedding bioinstructive 3D multilayered microchannels, developed by combining 3D printing with the layer-by-layer (LbL) assembly technology, in photopolymerizable hydrogels. Alginate (ALG) was chosen as the ink to produce customizable 3D sacrificial microstructures owing to its biocompatibility and structural similarity to the extracellular matrices of native tissues. ALG structures were further LbL coated with bioinstructive chitosan and arginine–glycine–aspartic acid-coupled ALG multilayers, embedded in shear-thinning photocrosslinkable xanthan gum hydrogels and exposed to a calcium-chelating solution to form perfusable multilayered microchannels, mimicking the biological barriers, such as the basement membrane, in which the endothelial cells were seeded, denoting an enhanced cell adhesion. The 3D constructs hold great promise for engineering a wide array of large-scale 3D vascularized tissue constructs for modular TERM strategies.

## 1. Introduction

The ability to bioengineer biomimetic 3D tissue-like biofunctional vascular constructs to accurately recreate living tissue-specific vascular architectures and their physicochemical, biomechanical and biological functions is the major and long-standing goal of bottom-up tissue engineering and regenerative medicine (TERM), aiming to replace, restore and/or regenerate damaged tissues and organs [1,2,3,4,5,6]. Conventionally, hydrogels are known as the gold standard 3D platforms for cell encapsulation, controlled therapeutics delivery and construction of 3D tissue-like cell-biomaterial scaffolds for bottom-up TERM, owing to their numerous appealing features, including biocompatibility, highly hydrated 3D environment, tunable physicochemical properties, mechanical similarity to native tissues and ease of implantation via minimally invasive procedures [7,8,9,10]. To date, functional tissues, such as cartilage, bladder and skin, have been successfully engineered and translated into the clinical practice by resorting to hydrogel-based constructs [11,12,13,14]. However, their effectiveness in sustaining cell viability is limited to microsized systems. Larger hydrogels (above ca. 200 μm) denote an inhomogeneous cell distribution and poor long-term and controlled diffusion of oxygen, nutrients, and metabolic waste removal due to their inability to build spatially organized perfusable vascular networks, thus leading to cell apoptosis and the formation of necrotic cores that dictate their failure upon implantation and prevent their clinical translation [15]. As such, engineering complex and large vascularized functional tissue constructs remains elusive, being essential to maintain tissue health.

Over the last two decades, significant progress has been made in the engineering of 3D natural and synthetic hydrogel-based scaffolds, embedding perfusable microchannel networks that are to be seeded with cells, which improve their mass transport properties to sustain long-term cell viability, and enable the formation of complex and large 3D vascularized tissue constructs by resorting to a wide variety of microfabrication techniques [16].

Among them, sacrificial molding techniques, including soft lithography and replica molding [17,18,19,20,21,22,23], and more recently 3D (bio)printing [24,25,26,27,28,29,30,31,32,33,34,35] or combinations of thereof [25,36,37,38], have been widely employed to engineer perfusable microfluidic networks of controlled sizes and geometries within hydrogel scaffolds for generating functional vascularized tissue-engineered constructs. However, the former is laborious, costly, time-consuming and difficult to scale-up. In addition, it is mostly applied to the fabrication of 2D planar perfusable microchannels that do not recreate the complex 3D tissue architecture, and most commonly resorts to the use of cytotoxic organic solvents or harmful processing conditions, including extreme temperature for removing the sacrificial templates, thus not being compliant with biomolecules. Moreover, despite the tremendous growth and exciting progress denoted by the 3D (bio)printing technology over the last few years, it still has limitations on printing resolution, choice of (bio)materials and living cells, controlled cell distributions and vascularization, and thus do not enable engineering 3D constructs across all scales and fully recreating the complexity of native tissues [39,40]. In contrast, the key enabling features endowed by the layer-by-layer (LbL) assembly technology turn it into a promising alternative to engineer bioinstructive perfusable microchannels exhibiting multifunctionalities. This technology has proven to be a simple, cost-effective, biologically safe and highly versatile bottom-up approach to conformally coat any type of surface and precisely engineer highly hydrated and hierarchical extracellular matrix-mimetic biomaterials, with fined-tuned structures, properties and functions at the nanoscale, by resorting to a myriad of biological components exhibiting complementary interactions [41,42,43,44,45,46,47,48,49,50]. However, the lack of a carrier platform that could encapsulate, protect and endow the developed biomaterial structures with superior biomechanical stability to be administered in the human body via minimally invasive procedures extensively limits the use of the LbL technology on its own.

Herein, we propose a new generation of bioengineered, cytocompatible and customizable 3D perfusable bioinstructive multilayered microchannels embedded in photopolymerizable natural-origin hydrogels for promoting the formation of microvascular networks in 3D engineered-tissue constructs in vitro, to be potentially used in modular TERM strategies. The cytocompatible freeform microchannels were prepared by 3D printing biocompatible alginate (ALG) sacrificial templates. ALG biopolymer was chosen as the ink to produce the customizable 3D printed structures due to its widely and readily availability, proven biocompatibility, non-cytotoxicity and non-immunogenic properties [51]. Moreover, the ionically crosslinked ALG core template can be easily liquefied, under mild conditions, using ethylenediaminetetraacetic acid (EDTA), thus turning it into a very appealing sacrificial template for being coated with bioinstructive multilayered films in a LbL fashion and engineer hollow multilayered nanostructures for fulfilling biomedical purposes. The 3D printed ALG sacrificial template structures were conformally LbL surface functionalized with bioinstructive multilayered thin films endowed with cell adhesion motifs, namely, chitosan (CHT)/arginine–glycine–aspartic acid (RGD)-grafted ALG bilayers. The LbL-coated ALG structures were further embedded in a shear-thinning, pseudo-plastic supporting pre-hydrogel matrix encompassing biocompatible and biodegradable glycidyl methacrylated xanthan gum (XG-GMA) [52], to engineer the bioactive and robust 3D constructs after UV light-induced photocrosslinking. The exposure of the 3D construct to an EDTA aqueous solution led to the liquefaction of the ALG permissive core, generating bioinstructive hollow multilayered microchannels embedded within the XG-GMA supporting hydrogel matrix for the culture of human umbilical vein endothelial cells (HUVECs). Previous works have proposed the use of the LbL method to recapitulate biological barriers, such as the basement membrane [53,54]. However, to the best of our knowledge, it is the first time that tubular structures made from multilayers are embedded in a supporting hydrogel matrix as perfusable microchannels. We hypothesize that this strategy could assign a variety of properties to the bioengineered construct, including mass transport and mechanical microenvironment control between the hydrogel and the microchannel, as well as exposure of biochemical factors (either on the surface or encapsulated in the multilayers) to the cells. The in vitro biocompatibility of the developed bioactive 3D constructs was studied, revealing an enhanced cell viability for HUVECs seeded on the bioactive LbL-functionalized microchannels, holding great promise for the generation of biofunctional vascular networks. The high versatility imparted by the combination of 3D printing, LbL assembly technology and photocrosslinkable hydrogels enables the fabrication of customizable and bioinstructive perfusable multilayered microchannels, potentially opening new avenues for controlled therapeutics delivery, as well as for engineering a wide array of large-scale 3D vascularized tissue constructs for modular TERM strategies.

## 2. Materials and Methods

### 2.1. Materials

Sodium ALG derived from brown algae (Mw = 538 kDa, viscosity ≈250 cP) was purchased from Sigma-Aldrich (St. Louis, MO, USA) and used as received. ALG-RGD (NOVATACH^TM^ MVG GRGDSP peptide-coupled ALG) was purchased from NovaMatrix (Sandvika, Norway) and used as received. CHT of medium molecular weight (Mw = 236.8 kDa, 80% degree of deacetylation, viscosity ≈390 cP) was kindly provided by Primex EHF (Siglufjordur, Iceland) and used without further purification. XG (2–20 MDa) was purchased from Doves Farm Foods Ltd. (distributed by Bricer Unipessoal, Portugal). Phosphate buffered saline (PBS), sodium hydroxide (NaOH), calcium chloride (CaCl_2_), EDTA, 2-hydroxy-4′-(2-hydroxyethoxy)-2-methylpropiophenone (Irgacure 2959), rhodamine B isothiocyanate (RITC), dispase II, collagenase type IV, Medium 199 (M199) and gelatin Type B from bovine skin were purchased from Sigma-Aldrich (St. Louis, MO, USA). Glacial acetic acid (CH_3_COOH) and hydrogen peroxide (H_2_O_2_) were purchased from JMGS (Odivelas, Lisboa, Portugal) and Carlo Erba (Sabadell, Barcelona, Spain), respectively. GMA (>95%, stabilized with hydroquinone monomethyl ether (MEHQ)) was purchased from TCI Chemicals (New Brunswick, NJ, USA). Heparin was purchased from PanReac AppliChem ITW Reagents (Darmstadt, Germany). All other reagents, namely ammonium hydroxide (NH_4_OH), dimethyl sulfoxide (DMSO), GlutaMAX™-I, fetal bovine serum (FBS), penicillin, streptomycin and live/dead kit were purchased from Thermo Fischer Scientific (Fair Lawn, NJ, USA). All the aqueous solutions were prepared using ultrapure water from a Milli-Q Plus water purification system (resistivity >18.2 MΩ cm) from Merck Millipore (Burlington, MA, USA).

### 2.2. Preparation of ALG Ink

An ALG ink was prepared following the method described by Freeman and Kelly, with slight changes in the ink viscosity [55]. Briefly, an ALG ink was prepared at 5% (*w/v*) in PBS. Moreover, a 60 mM CaCl_2_ aqueous solution was prepared to ionically pre-crosslink the ALG ink by mixing the two solutions at a volumetric ratio (v/v) of 25:9 (ALG solution:CaCl_2_ solution) for 30 min. Then, the ALG ink was ready to be printed.

### 2.3. 3D Printing of ALG Ink

3D solid ALG microstructures were designed with computer-aided design (CAD) software (SolidWorks Student Standard (SSS) 2020). ALG structures with different sizes and geometries were 3D printed using a 23G needle tip (diameter = 330 µm). The size of the ALG structures ranged from 1 to 5 cm, according to the complexity of the ALG structures to be printed. Afterwards, the CAD designs were assembled using 3D printing software (Repetier-Host 2.0.5) and, following the axes calibration, the previously ALG ink-loaded cartridges were fitted and placed into the printer’s cartridge holder. The printing process was performed at room temperature, with an extrusion pressure of 50 kPa and a needle speed of 25 mm s^−1^ using an Inkredible+ 3D bioprinter (Cellink, Gothenburg, Sweden). Then, the 3D printed ALG designs were immersed in a 60 mM CaCl_2_ aqueous solution for 5 min to ionically post-crosslink the ALG structures followed by rinsing with ultrapure water. 

### 2.4. Synthesis of RITC-Labeled CHT

RITC-labeled CHT was synthesized by preparing a 1% (w/v) CHT aqueous solution in 0.1 M acetic acid, followed by the addition of anhydrous methanol under stirring for 3 h. The reaction proceeded for 4 h in the dark at room temperature. Then, the RITC-labeled CHT was precipitated in 0.2 M NaOH and washed with methanol (70%) until the supernatant was fluorescence-free. RITC-CHT was dissolved in 0.1 M acetic acid and dialyzed against deionized water for 7 days using a 6–8 kDa MWCO regenerated cellulose membrane (Spectrum Laboratories Inc., Rancho Dominguez, CA, USA). The whole reaction and dialysis purification process were performed protected from light. Finally, RITC-CHT was freeze-dried and stored at 4 °C until use.

### 2.5. Zeta (ζ)-Potential Measurements

Before the build-up of the multilayered thin films, the electrophoretic mobility of the freshly prepared 0.5 mg mL^−1^ CHT, ALG and ALG-RGD aqueous solutions (pH 5.5) was studied by measuring their zeta (ζ)-potentials. The ζ-potentials of the individual solutions were measured using a Zetasizer Nano-ZS (Malvern Instruments Ltd., Royston, Hertfordshire, UK) at 25 °C. The electrophoretic mobility (*u*) was converted into a ζ-potential value following the Smoluchowski equation (*ζ* = *uη/ε*, where *η* and *ε* stand for the viscosity and permittivity of the solution, respectively) [56]. The measurements were performed in triplicate and averaged for each sample.

### 2.6. Quartz Crystal Microbalance with Dissipation Monitoring (QCM-D)

The growth of the bioactive, electrostatic-driven CHT/ALG-RGD multilayered thin films onto the gold-coated 5 MHz AT-cut quartz crystal sensors (QSX301 Gold, Q-Sense, Sweden) was monitored in situ by QCM-D (QSense Pro, Biolin Scientific, Gothenburg, Sweden). Before that, the gold-coated quartz crystal substrates were thoroughly cleaned with an oxidizing cleaning solution encompassing a 1:1:5 (v/v) mixture of NH_4_OH (25%), H_2_O_2_ (30%) and ultrapure water in an ultrasonic bath at 70 °C for 10 min. The Au-plated quartz crystal sensors were then thoroughly rinsed with ultrapure water at room temperature, dried under a soft stream of N_2_ and submitted to UV/Ozone (UV/Ozone ProCleaner 220, BioForce Nanosciences, Inc., Ames, IA, USA) treatment for 10 min. The freshly cleaned sensors were inserted in the QCM-D apparatus and equilibrated in an aqueous solution (pH 5.5) until a stable baseline was achieved. Afterwards, the gold-plated quartz sensors were alternately exposed to a 0.5 mg mL^−1^ CHT (6 min adsorption time) and RGD-coupled ALG (6 min adsorption time) aqueous solutions at pH 5.5, rendering the substrate surface positively and negatively charged, respectively. In-between the deposition of the oppositely charged biopolymeric materials, the substrates were rinsed with an aqueous solution (pH 5.5) for 4 min to remove loosely adsorbed molecules. The assembly process was repeated six times until reaching (CHT/ALG-RGD)_6_ multilayered thin films. The final (CHT/ALG-RGD)_6_ multilayered thin films were dried under a soft stream of N_2_. The experiments were performed at a constant flow rate of 50 μL min^−1^ and at 25 °C. The gold-plated quartz crystal substrates were excited at multiple overtones (1, 3, 5, 7, 9 and 11 corresponding to 5, 15, 25, 35, 45 and 55 MHz, respectively) and the changes in the frequency (Δ*f*) and dissipation (Δ*D*) were monitored in real time. Moreover, the frequency of each overtone was normalized to the fundamental resonant frequency (5 MHz) of the quartz crystal substrate (Δ*f_n_/n*, *n* refers to the overtone number).

The hydrodynamic thickness of each of the adsorbed layers was estimated using the Voigt-based viscoelastic model implemented in the Q-Sense Dfind software (Broadfit function), assuming a fluid viscosity of 1 mPa s and a fluid and layer density of 1000 kg m^−3^.

### 2.7. Build-Up of 3D Printed ALG-Templated Bioinstructive Polysaccharide-Based Multilayered Thin Films

The build-up of the bioactive (CHT/ALG-RGD)_6_ multilayered thin films onto the 3D printed ALG-templated microstructures was performed by repeating the LbL alternate immersion of the ALG-printed sacrificial structures in 1 mg mL^−1^ CHT and ALG-RGD aqueous solutions (pH 5.5) for 6 min each using a home-made automatic dipping robot (CORPUS^®^, Guimarães, Portugal). In-between the deposition of the oppositely charged biopolymers, the ALG-templated microstructures were rinsed with an aqueous solution at pH 5.5 for 4 min to remove the loosely adsorbed molecules and avoid the cross-contamination of the biopolymeric solutions. The deposition cycles were repeated six times until reaching a (CHT/ALG-RGD)_6_ multilayered film. By the end of the assembly process, the bioinstructive (CHT/ALG-RGD)_6_ LbL-functionalized, 3D printed ALG microstructures were kept in water at 4 °C until used, to prevent them from drying and collapsing. The preparation of the (RITC-CHT/ALG)_6_ multilayers templated on 3D printed ALG sacrificial templates was performed in a reminiscent fashion, aiming to prove the successful and conformal LbL coating by fluorescence microscopy (Axio Imager M2 upright widefield fluorescent microscope, Carl Zeiss, Jena, Germany).

### 2.8. Preparation of Bioactive 3D Constructs

Bioactive 3D constructs encompassing bioinstructive (CHT/ALG-RGD)_6_ multilayered films functionalized 3D printed ALG sacrificial microstructures embedded in photocrosslinkable XG-GMA supporting hydrogel matrices were prepared, preventing the collapsing of the LbL-functionalized microstructure after ALG core template liquefaction and the culture of HUVECs in the hollow microchannels to engineer prevascular networks.

#### 2.8.1. Synthesis of XG-GMA

The chemical modification of the XG polymer backbone with photopolymerizable methacrylate groups was performed as previously described [57,58]. Briefly, a 0.5% (w/v) XG aqueous solution was prepared in deionized water, under vigorous stirring, at room temperature. GMA (5 mL) was added to this solution dropwise and the mixture was stirred for 12–18 h at 80 °C. Then, the unreacted molecules were removed by dialysis against deionized water for 3 days, at room temperature and in the dark, using a 6–8 kDa MWCO regenerated cellulose membrane (Spectrum Laboratories Inc., Rancho Dominguez, CA, USA). Finally, the resulting solution was frozen at −80 °C and freeze-dried (Telstar LyoQuest Plus Eco, VWR). The lyophilized XG-GMA was stored at 4 °C, in the dark, until use. The chemical characterization of the obtained XG-GMA compound has been previously reported by our group, confirming the effective functionalization of the XG biopolymer backbone with GMA [58].

#### 2.8.2. Preparation of Photocurable XG-GMA Pre-Hydrogels

The photocurable XG-GMA pre-hydrogel aqueous solution was prepared by solubilizing lyophilized XG-GMA in PBS (pH 7.4) containing 0.1% (w/v) of Irgacure 2959 to a final concentration of 0.5% (w/v).

#### 2.8.3. Preparation of Photocrosslinkable XG-GMA Hydrogels Embedding Perfusable Microchannels

The obtained XG-GMA pre-hydrogel aqueous solutions were poured into a silicone mold and the 3D printed ALG template sacrificial microstructure, either non-functionalized or LbL functionalized with the bioinstructive (CHT/ALG-RGD)_6_ multilayered thin film, was embedded on it, leaving both ends of the ALG microstructure outside the XG-GMA pre-hydrogel solution at opposing ends. Then, the XG-GMA pre-hydrogel solution was crosslinked by exposure to UV light irradiation (OmniCure S2000, 320–500 nm, 100 mW cm^−2^, Mississauga, ON, Canada) for 60 s. The photocrosslinkable XG-GMA hydrogel, embedding either the uncoated or the bioactive LbL-functionalized 3D printed ALG-templated microstructure, was immersed in a 10 mM EDTA aqueous solution (pH 8) overnight to liquefy the 3D printed ALG core structure and obtain a hollow multilayered microchannel within the XG-GMA hydrogel.

#### 2.8.4. Rheological Characterization of the Photocrosslinkable XG-GMA Hydrogels

The rheological characterization of the XG-GMA pre-hydrogel supporting matrix at 0.5% (w/v) was performed at 25 °C on a Kinexus Lab+ rheometer (Malvern Panalytical, Malvern, UK), equipped with a UV curing chamber fitted with a 20 mm-diameter parallel plate geometry and a gap size of 500 µm. The shear-thinning properties were studied by measuring the variation in the viscosity with a continuously ramped shear rate (0.1 to 100 s^−1^). The linear viscoelastic region (LVER) was determined by measuring the strain amplitude sweep (0.1 to 1000%) at a frequency of 1 Hz. Oscillatory frequency sweep testing (0.01 to 10 Hz) was conducted at 1% strain amplitude. To demonstrate the variation in the XG-GMA elastic modulus during the photocrosslinking process, a time sweep measurement was carried out at 1 Hz with UV light exposure (OmniCure S2000, 320–500 nm, 100 mW cm^−2^, Mississauga, ON, Canada).

### 2.9. In Vitro Cell Culture

#### 2.9.1. Cell Isolation and Culture

HUVECs were retrieved from umbilical cord obtained under a cooperation agreement established between CICECO – Aveiro Institute of Materials, University of Aveiro and Centro Hospitalar do Baixo Vouga (Aveiro, Portugal), after approval by the Competent Ethics Committee (CEC). The received human tissues were handled in accordance with the guidelines approved by the CEC and informed consent was obtained from all subjects. HUVECs were isolated following an well-established protocol in the group [59]. Briefly, an enzymatic mixture containing dispase II and collagenase type IV was used to isolate the HUVECs from the umbilical cord. The cord vein was filled with the enzyme cocktail. Multiple site injections out in the cord matrix using the enzyme cocktail were also carried. Subsequently, the cord was incubated at 37 °C for 20 min, and then the HUVECs were seeded in M199 growth medium followed by their incubation in a humidified atmosphere of 5% CO_2_ at 37 °C. After 4–6 h, the medium was replaced by fresh M199 containing 20% umbilical cord blood serum, 2 mM L-glutamine, 5 ng mL^−1^ vascular endothelial growth factor, 10 μg mL^−1^ heparin, 100 U mL^−1^ penicillin, and 100 μg mL^−1^ streptomycin [60].

Prior to HUVECs culture, a T75 cell culture flask was coated with a 2% gelatin solution and left to incubate for at least 30 min, at 37 °C, in a humified atmosphere containing 5% CO_2_. Then, the gelatin layer was cleaned with sterile PBS. Cells were seeded in the pre-coated T75 cell culture flask at a density of 1 × 10^6^ cells/mL and incubated at 37 °C in a humified atmosphere containing 5% CO_2_. The culture medium was changed every 2–3 days. Cell proliferation was monitored using a light microscope (Axiocam 105 color, Carl Zeiss, Jena, Germany).

#### 2.9.2. Cell Seeding and Viability within the Perfusable Microchannels Embedded in 3D Printed XG-GMA Hydrogels

Upon reaching the confluence, HUVECs were trypsinized. New cell suspensions were prepared at a density of 2 × 10^6^ cells/mL in M199 culture media supplemented with and without FBS. Then, cells were seeded into either the LbL-free or (CHT/ALG-RGD)_6_ LbL-functionalized hollow microchannels embedded in the XG-GMA hydrogels for 4 h at 37 °C in a humified 5% CO_2_ atmosphere to allow endothelial cells to adhere to the microchannels. To evaluate the cell viability after 3 days of culture, the XG-GMA hydrogels embedding either the uncoated or (CHT/ALG-RGD)_6_ LbL-functionalized hollow microchannels were incubated in a calcein-AM/propidium iodide (PI) solution for 30 min. The overall 3D constructs were imaged using confocal laser scanning microscopy (CLSM; LSM 880 Airy Scan, Zeiss, Jena, Germany). The acquired data were processed in Zeiss ZEN v3.0 blue edition software. All HUVECs used were between passages 5 and 7 to ensure the representation of key endothelial cell characteristics. The quantification of the cell viability was performed using the ImageJ software (Fiji 1.52n).

## 3. Results and Discussion

**Figure 1:**
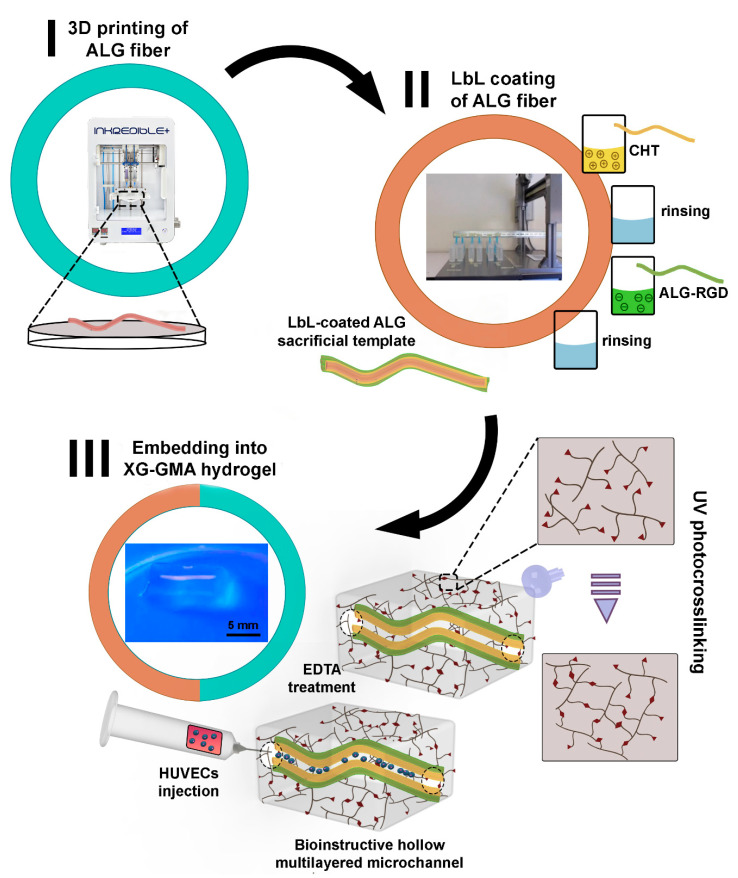
Schematic illustration of the procedure behind the fabrication of 3D perfusable constructs encompassing bioinstructive (CHT/ALG-RGD)_6_ multilayers templated on liquefied ALG microchannels embedded in photocrosslinkable XG-GMA supporting hydrogels.

In this work, bioactive 3D constructs encompassing (i) bioinstructive LbL-functionalized 3D printed multilayered microchannels embedded in (ii) biocompatible and biodegradable photocrosslinkable XG-GMA hydrogels were developed aiming for prevascular networks. Briefly, biocompatible ALG core microstructures exhibiting different sizes and geometries were successfully 3D printed. Such structures were further LbL functionalized with multilayers encompassing biocompatible, non-cytotoxic, non-immunogenic and readily available marine-origin polysaccharides, namely, positively charged CHT and negatively charged ALG modified with the cell-adhesive peptide sequence RGD. The bioinstructive LbL-coated ALG microstructures were further embedded within a supporting biomaterial consisting of XG-GMA, which, after photocrosslinking and immersion in an EDTA aqueous solution, enabled engineering the hollow microchannels. Then, HUVECs were seeded onto the microchannels aiming to colonize them and form prevascular networks (Figure 1).

### 3.1. Fabrication of 3D Printed ALG Sacrificial Template Structures

**Figure 2:**
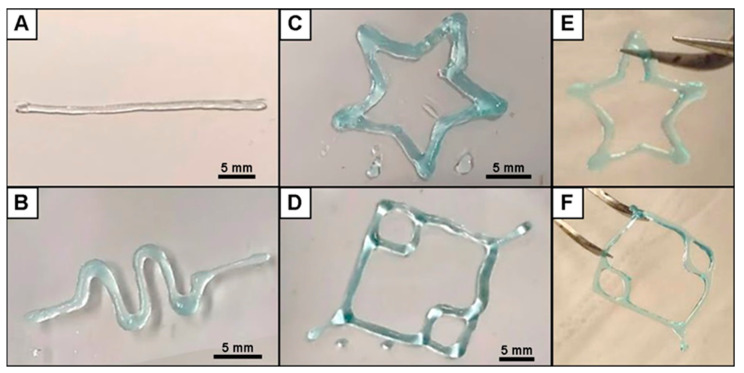
Optical images of the 3D printed ALG sacrificial template structures, exhibiting their different sizes and shapes, after crosslinking with CaCl_2_: (**A**) cylinder, (**B**) sinusoidal, (**C**) star and (**D**) capillary structures. (**E**) Star and (**F**) capillary 3D printed photographs, showing their handling ability.

Customizable 3D ALG sacrificial template structures exhibiting different sizes and shapes, from simple to more complex structures, have been designed by the CAD model and 3D printed, revealing the high versatility imparted by the 3D printing technology (Figure 2). The 3D printed ALG structures were strong enough to sustain handling and maintain their shape, as shown in Figure 2E,F, thus holding great promise for the design and development of more intricate and personalized artificial biological architectures to be used in a wide array of application scenarios, including in controlled therapeutics delivery, biosensing, tissue engineering and regenerative medicine strategies.

### 3.2. Build-Up of Bioinstructive CHT/ALG-RGD Multilayered Thin Films on 2D and 3D Surfaces

Prior to the LbL surface functionalization of the 2D quartz crystal sensors and 3D printed ALG-templated sacrificial structures with bioinstructive (CHT/ALG-RGD)_6_ bilayers, the net electrical charge of freshly prepared CHT and RGD-coupled ALG aqueous solutions at the working pH of 5.5 was assessed by measuring their ζ-potentials. The ζ-potentials of ALG (core template), ALG-RGD and CHT aqueous solutions at pH 5.5 were found to be −23.4 ± 1.6 mV, −19.7 ± 2.7 mV and +17.9 ± 0.4 mV, respectively, thus unveiling the anionic nature of ALG and ALG-RGD (pH > p*K*_a_ ~3.38 or 3.65 for mannuronic or guluronic acid residues, respectively) [51,61], and the cationic nature of the CHT biopolymeric solutions (pH < p*K*_a_ ~6–6.5) [62]. As such, we hypothesize that the positively charged CHT and the negatively charged ALG-RGD biopolymers could successfully build-up electrostatic-driven multilayered thin films by exploiting the attractive electrostatic interactions between the oppositely charged biopolymers. Hence, the possible build-up of 2D CHT/ALG-RGD multilayered thin films via electrostatic interactions between the oppositely charged biocompatible CHT and RGD-coupled ALG biopolymers was assessed in situ by the QCM-D technique by applying an alternating electric field across the gold-coated quartz crystal sensor [63]. The QCM-D technique allows us to detect very small changes in the hydrodynamic mass (ng cm^−2^) due to changes in the resonance frequency of the quartz crystal sensor and measure the viscoelastic properties of the adsorbed layers through the energy dissipated in the mechanical oscillation of the quartz sensors [63,64].

**Figure 3:**
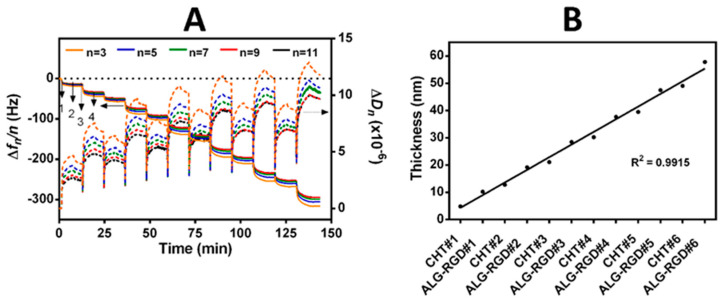
Build-up of (CHT/ALG-RGD)_6_ multilayered thin films onto Au-plated quartz crystal sensors. (**A**) QCM-D monitoring of the normalized frequency (Δ*f_n_/n*) and dissipation (Δ*D_n_*) shifts, obtained at the 3rd (*n* = 3; 15 MHz), 5th (*n* = 5; 25 MHz), 7th (*n* = 7; 35 MHz), 9th (*n* = 9; 45 MHz) and 11th overtones (*n* = 11; 55 MHz), as a function of time for the build-up of (CHT/ALG-RGD)_6_ bilayers onto gold-plated quartz crystal sensors and intermediate rinsing steps. The numbers refer to the adsorption of CHT (1), ALG-RGD (3) and rinsing steps (2 and 4). (**B**) Cumulative hydrodynamic thickness evolution for the (CHT/ALG-RGD)_6_ multilayered thin films, estimated using the Voigt-based viscoelastic model. The black straight line represents the linear regression fit accompanied by the respective coefficient of determination (*R*^2^ = 0.9915).

Figure 3A showcases the normalized frequency (Δ*f_n_*/*n*) and dissipation factor (Δ*D_n_*) changes obtained at the 3rd (*n* = 3; 15 MHz), 5th (*n* = 5; 25 MHz), 7th (*n* = 7; 35 MHz), 9th (*n* = 9; 45 MHz) and 11th overtones (*n* = 11; 55 MHz) during the build-up of multilayered thin films, encompassing six CHT/ALG-RGD bilayers, onto the gold-plated quartz crystal substrate.

The sequential decrease in the Δ*f_n_/n* after the adsorption of each biopolymeric aqueous solution, i.e., CHT and ALG-RGD, onto the Au-plated quartz crystal surface proves the deposition and effective interaction of the deposited materials throughout the deposition cycles.

Moreover, the successive increase in the Δ*D_n_* after each deposition step together with the separation denoted by the different overtones, mainly for the latest adsorbed bilayers, reveal the viscoelasticity of the adsorbed layers, which are not constant throughout the adsorption process. This is a typical feature of soft and hydrated polymeric films [65]. In overall, the decrease in Δ*f_n_/n* and the increase in the Δ*D_n_* values suggest that the negatively charged ALG-RGD molecules are adsorbed onto the positively charged CHT layer, confirming the electrostatic-driven interaction between both biopolymers and the stable step-by-step growth of the LbL assemblies. In addition, the rinsing steps did not induce changes in the Δ*f_n_/n* and Δ*D_n_* data after the deposition of each layered material, thus confirming the strong interaction between the biopolymers, as well as the irreversible nature of the adsorption process.

The hydrodynamic thickness of the multilayered film per adsorbed layer was also estimated using the Voigt-based viscoelastic model [66], as showcased in Figure 3B, revealing the nanostructured dimension of the adsorbed layers. Furthermore, a linear increase in the hydrodynamic thickness along the adsorption cycles was observed, corroborating the increase in the −Δ*f_n_/n* and Δ*D_n_* values. The build-up of the (CHT/ALG-RGD)_6_ multilayered thin film led to a final multilayered thickness of ca. 58 nm.

**Figure 4:**
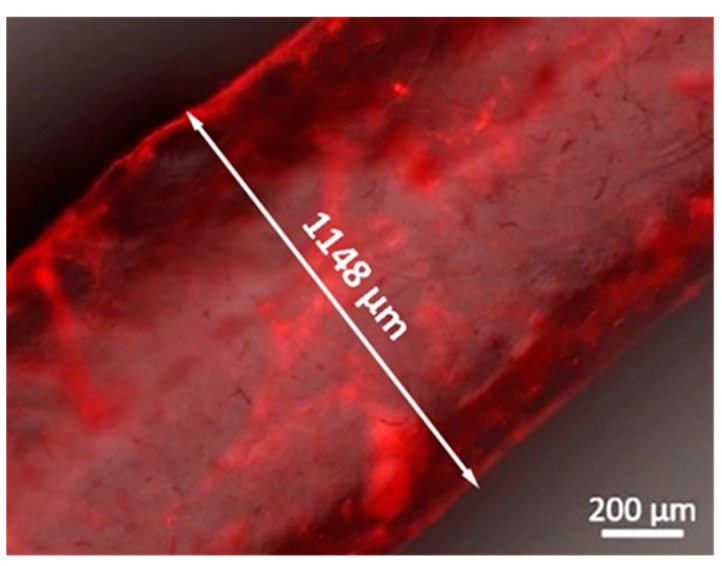
Representative fluorescence microscopy image of a 3D printed ALG sacrificial microfiber coated with (RITC-CHT/ALG)_6_ multilayered thin films in a LbL fashion.

Following the successful interaction and the stable LbL growth of the bioactive (CHT/ALG-RGD)_6_ multilayered thin films on 2D quartz crystal sensors, similar multilayered thin films have been successfully and conformally templated on 3D printed ALG sacrificial microfiber templates (ca. 1150 μm in diameter, corroborating a previous report [29]), using an automatic dipping robot, as confirmed by labeling the CHT biopolymer with RITC (Figure 4). Although the effective LbL coating has been demonstrated for the 3D printed ALG fiber-like structures, the high versatility imparted by the LbL assembly technology enables coating virtually any type of template, regardless of its size, shape, and surface chemistry, thus opening new avenues in modular tissue engineering. Previously, we have demonstrated such versatility by coating ALG hydrogel particles with a similar multilayered thin coating [67,68,69]. In addition, we have demonstrated that a minimum of six bilayers was needed to produce a mechanically stable, robust, and conformal core-shell structure that could sustain its manipulation and implantation [68], thus being the rationale behind coating the 3D printed ALG structures with six bilayers. Moreover, we have also validated previously the thin multilayered nanocoating as a permselective membrane, enabling the diffusion of essential molecules for cell survival, such as nutrients and oxygen, and the exchange of metabolites and waste products.

### 3.3. Fabrication and Characterization of Perfusable Hollow Tubular 3D Constructs

Bioactive 3D constructs comprehending 3D printed LbL-functionalized bioinstructive hollow microchannels embedded in biocompatible XG-GMA supporting hydrogels were bioengineered aiming to seed HUVECs within the inner walls of the hollow microchannels and stimulate the formation of prevascular networks.

**Figure 5:**
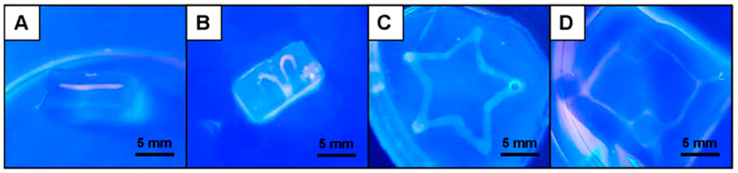
Optical images of the customizable 3D constructs enclosing perfusable microchannels embedded in photocrosslinkable XG-GMA hydrogels after the injection of a fluorescent aqueous solution into the microchannel inner walls, viewed under UV light: (**A**) cylinder, (**B**) sinusoidal, (**C**) star and (**D**) capillary-like structures.

XG-GMA hydrogels were easily and quickly prepared on silicone molds by photopolymerization upon exposure of their pre-hydrogel aqueous solutions, embedding either the uncoated or bioinstructive (CHT/ALG-RGD)_6_ functionalized ALG sacrificial template, to UV light irradiation (100 mW cm^−2^) for 60 s. The successful development of photocrosslinkable XG-GMA hydrogels was proven by the ability of the hydrogels to retain its printed shape upon the silicone mold removal. Then, the full 3D construct was immersed in an EDTA calcium-chelating aqueous solution at pH 8 to liquefy the 3D printed ALG core sacrificial template, leading to a perfusable, hollow, multilayered channel whose shape was permanently fixed and sustained without collapsing by the shear-thinning pseudo-plasticity showcased by the XG-GMA supporting hydrogel matrix. In fact, XG was selected as the biomaterial for the preparation of the hydrogel supporting matrix owing to its very high viscosity at a very low biopolymer concentration, assigning outstanding suspending properties [52]. To evaluate the perfusion extension of the hollow microchannel embedded within the hydrogel, a fluorescent dye aqueous solution was injected into the inner walls of the hollow multilayered microchannel (Figure 5; Video S1, Supplementary Materials). The successful flow of the fluorescent dye solution through the inner walls of the multilayered microchannel embedded in the photocrosslinkable XG-GMA hydrogel was seen by naked eye, thus revealing that the 3D printed ALG sacrificial template was effectively liquified and could be populated with endothelial cells to engineer endothelial cell-lined tubular networks.

### 3.4. Rheological Characterization of XG-GMA Hydrogel

**Figure 6:**
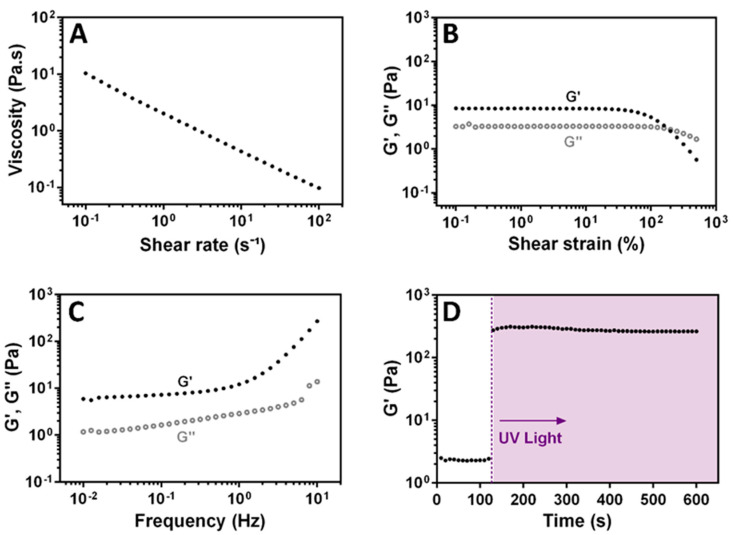
Rheological characterization of the 0.5% (w/v) XG-GMA pre-hydrogel aqueous solution: (**A**) shear viscosity with increasing shear rates (0.1–100 s^−1^), (**B**) elastic modulus (G′, filled circle) and viscous modulus (G″, open circle) as a function of strain (0.1–1000%, 1 Hz), (**C**) frequency sweep at a strain of 1% (0.01–10 Hz) and (**D**) elastic modulus of the photocrosslinked XG-GMA hydrogel, supplemented with 0.1% (w/v) photo-initiator, after UV light exposure (shaded area).

The shear-thinning behavior of a 0.5% (w/v) XG-GMA pre-hydrogel aqueous solution was studied (Figure 6). Despite the very low concentration, the increase in the shear rate led to a decrease in the viscosity, following the power law model, as denoted by Figure 6A. This result confirms that the XG-GMA preserves the shear-thinning behavior of the native natural-origin XG biopolymer, fixing the microchannel without falling. As shown in Figure 6B, both elastic (G’) and viscous (G’’) moduli are strain independent over a large LVER, which is indicative of a well-dispersed and stable system. Additionally, to verify the gel-like properties of the XG-GMA supporting matrix, a frequency sweep at low strain amplitude (1%) was conducted (Figure 6C). The gel-like XG-GMA matrix exhibits a minor frequency-dependent viscoelastic behavior, with the G’ dominating over G’’. The photo-gelling response of the XG-GMA hydrogel was analyzed under UV light exposure, denoting a drastic increase of the G’ values, which stabilized within 60 s. The increase in the G’ values by two orders of magnitude corresponds to the transition of the XG-GMA gel-like behavior to an elastic hydrogel (Figure 6D). The hydrogel formation enabled shape fixation and sustained the bioinstructive multilayered microchannel without collapsing after liquefying the ALG core sacrificial template.

### 3.5. In Vitro Biological Performance

**Figure 7:**
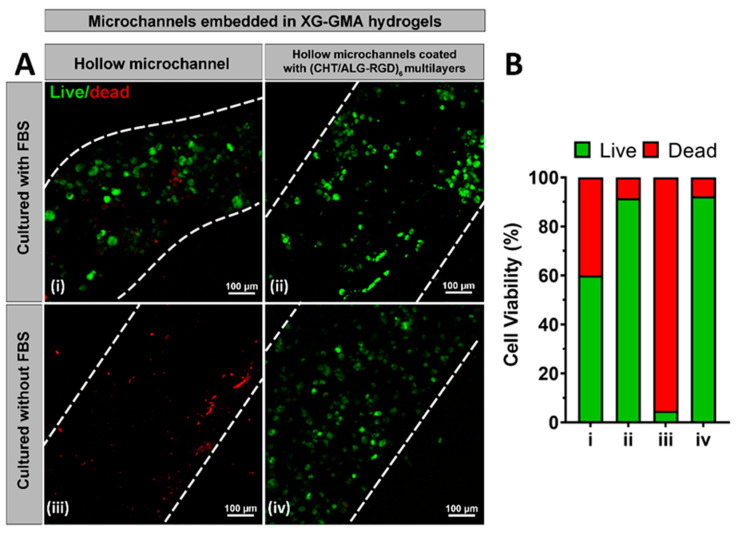
(**A**) Live/dead CLSM micrographs of HUVECs seeded for 3 days, with (**i**,**ii**) or without (**iii**,**iv**) FBS, in the uncoated (**i**,**iii**) and (CHT/ALG-RGD)_6_-coated hollow microchannels (**ii**,**iv**) embedded in photocrosslinkable XG-GMA hydrogels. White dashed lines indicate the boarders of the microchannels. (**B**) Quantification of cell viability in the uncoated and LbL-coated microchannels for HUVECs seeded for 3 days with or without FBS.

The HUVECs’ viability within the inner walls of either the uncoated or bioinstructive (CHT/ALG-RGD)_6_ LbL-coated perfusable microchannels embedded in photocrosslinkable XG-GMA hydrogels was assessed via CLSM by performing a live/dead assay (Figure 7A) and quantified using ImageJ (Figure 7B), as described elsewhere [70]. Data was acquired at day 3 since no significant differences were found 24 h after seeding (data not shown). After seeding the HUVECs, using culture medium supplemented with FBS, into the inner walls of the perfusable microchannels, the photocrosslinkable XG-GMA hydrogel containing the microchannel functionalized with (CHT/ALG-RGD)_6_ multilayers revealed an increase in the number of viable cells (90%) when compared to the XG-GMA hydrogel embedding the LbL-free microchannel (60%, Figure 7A,B). However, a more pronounced and significant difference was seen while performing an FBS-free culture of HUVECs in the hollow microchannels. Less than 5% of the viable cells were observed on the non-functionalized microchannel whereas over 90% of viable cells could be seen on the microchannel coated with (CHT/ALG-RGD)_6_ multilayers. These results reveal the positive effect of the presence of the RGD peptide on the multilayered thin film on enhancing cell adhesion. In fact, RGD is well-known to be specifically recognized by the integrin receptors on the cell membrane as an adhesion domain, boosting both the cell adhesion and proliferation [71,72]. Our results highlight the potential of using the smooth inner walls of the microchannels functionalized with (CHT/ALG-RGD)_6_ multilayers in triggering the adhesion, proliferation, and migration of HUVECs and formation of endothelial cell-lined tubular networks when embedded in hydrogels, by providing the microchannel networks with cell-binding domains while allowing the exchange of oxygen and nutrients intrinsically needed for cell survival. It is important to highlight that this proof-of-concept work was performed with a hydrogel that does not exhibit any particular adhesive characteristics to HUVECs, to demonstrate the benefits of the bioactive, multilayer-coated microchannels. We hypothesize that the proposed methodology could be universally applied to improve the interaction with any cell type in microchannels embedded in virtually any type of hydrogel.

## 4. Conclusions

In this work, we have successfully developed 3D constructs encompassing bioinstructive LbL-coated customizable 3D printed perfusable microchannels embedded in photocrosslinkable hydrogels for vascular tissue engineering. The functionalization of the 3D printed ALG sacrificial microstructures with bioinstructive (CHT/ALG-RGD)_6_ multilayered thin films, and their further encapsulation in shear-thinning photocrosslinkable XG-GMA supporting hydrogels was demonstrated. The formation of bioinstructive perfusable multilayered microchannels was effectively unveiled, under mild conditions, by the liquefication of the ALG core template in EDTA. The impact of the multilayered nanocoating, displaying cell adhesion domains, on the seeded HUVECs was shown by the higher number of adherent and viable cells in the inner walls of the LbL-functionalized microchannels when compared with the uncoated ones, holding great promise for the development of endothelial cell-lined tubular networks as blood vessel substitutes. Although the field is still in its infancy, we hypothesize that the versatility imparted by the integration of 3D printing and LbL assembly technology in hydrogels could move a step forward and potentially lead to the design and development of microstructures with enhanced complexity and multiple biofunctionalities in a near future. We foresee that such structures could open new avenues in controlled therapeutics delivery, as well as in shaping a wide array of 3D constructs that could more closely emulate the hierarchical structure, organization and biofunctionality of living tissues.

## Data Availability

The data presented in this study are available within the article.

## Supplementary Materials

The following is available online at https://www.mdpi.com/article/10.3390/biom11060863/s1, Video S1: 3D perfusable bioinstructive multilayered microchannel embedded in photocrosslinkable natural-origin hydrogel showing the flow of a blue-fluorescent dye through the microchannel.
